# Machine learning–driven data perturbation techniques for privacy-preserving data mining

**DOI:** 10.1038/s41598-026-52200-8

**Published:** 2026-06-06

**Authors:** Nadella Sunil, G Narsimha

**Affiliations:** 1 Department of Computer Science & Engineering, UCEK, JNTUK Kakinada, Aditya University, Surampalem, Surampalem, East Godavari District, Kakinada, Andhra Pradesh 533437 India; 2 Department of Computer Science & Engineering, UCEJ, JNTUH, Telungana, India

**Keywords:** Privacy preserving data mining, Data perturbation, Flip and rotation perturbation, Analytic hierarchy process, Classification, Engineering, Mathematics and computing

## Abstract

The emergence of digital information has raised many issues over the release of sensitive personal information in data mining activities due to the rapid expansion of the digital information. Privacy-Preserving Data Mining (PPDM) has the goal of allowing a significant analysis of data, as well as safeguarding confidential characteristics. Conventional privacy methods, like anonymization usually decrease the usefulness of data, and cryptographic methods are expensive to compute. This paper suggests a perturbation-based PPDM model, which will combine the K-means + +  clustering algorithm with the Flip-and-Rotation Perturbation (FRP) algorithm and decision model based on the Analytic Hierarchy Process (AHP). The suggested solution would take the dimensionality of features before perturbation to ensure increased privacy and maintain classification value. Experimental testing to check on the proposed method proves that it has better performance in accuracy, precision, recall and the F-measure compared to the conventional Naive Bayes and fuzzy-based methods. These findings do confirm that the proposed framework is a useful trade-off in the balance of data utility and protection of privacy in structured datasets.

## Introduction

The growing danger of the Internet phishing^1–3^, introduces difficulties to the transmission of data with security and highlights essential character of privacy protection in data mining processes. This has created the privacy-preserving data mining (PPDM) to overcome the problem of allowing data analysis to be effective besides protecting sensitive data^4–6^. Industries, government, finance, healthcare and many others have been gathering large volumes of consumer information to analyze resulting in gigabytes of complex information, popularly referred to as big data^7,8^. The name of this activity is data mining which is an interdisciplinary computer programming branch. The concept of deep learning based on data (KADD) that is also known as data mining has been substituted with what is termed as information extraction as a title of explanation. The first one is the data preparation and the process involves selecting the data, cleaning the data (to remove the noise and to remove data redundancy), transforming the data (to integrate data collected by different sources); Data processing involves integrating and transforming data into mining-friendly forms; In the third step which is known as the mining, cognitive methods are applied towards extracting data sequences (such as classifications and clusters); Basic operations (i.e., pattern assessment and knowledge presentation): this stage involves identifying interesting patterns which can be considered as knowledge representations. These basic steps also entail the display of extracted information in a form that is understood. The second item of the process of data mining is data alteration. In order to achieve maximum levels of privacy and security, data manipulation is normally utilized to modify the original contents of a dataset to be availed to the open^9,10^. One form of data manipulation should be in balance with the privacy statement adopted in an organization. There are methods of changing data, which are:

- Blocked is the replacement of an existing attribute value by a "?". Perturbation refers to changing the value of an attribute using a new value (a 1-value changed to a 0-value or noise).
- Consolidation or, in other words, the combination is the procedure that involves aggregating numerous values into more coarse ones.
- Samples, which is associated with the mere revelation of data of a section of a population, and Swapping, which is also associated with the alteration of the figures of separate recordings.

PPDM has developed to deliver trustworthy data mining results and at the same time prevents privacy violations that sensitive data entails.

**Fig. 1:**
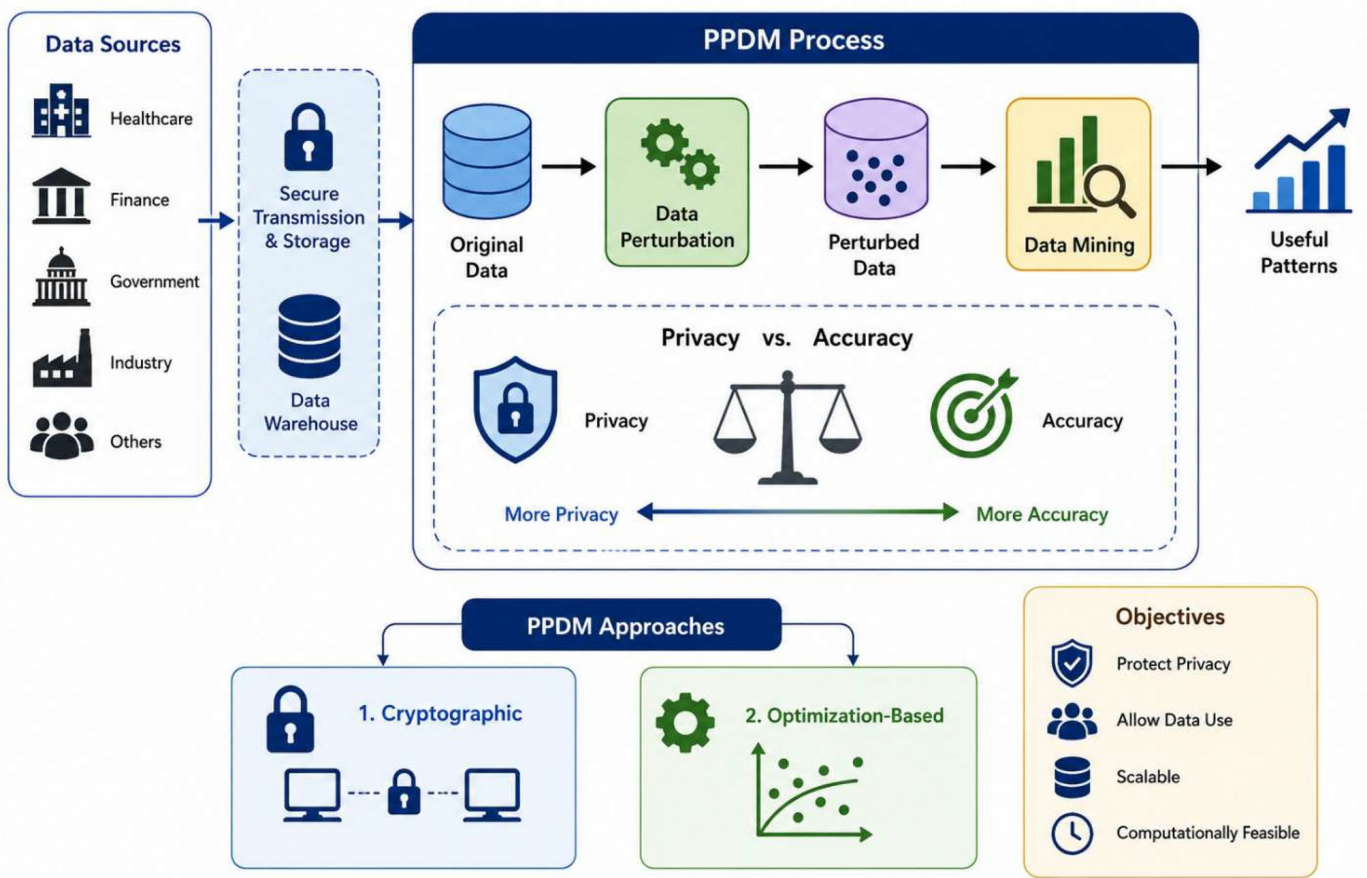
PPDM Approaches.

Figure 1 presents a distributed privacy preserving data mining (PPDM) case highlighting on privacy and accuracy. In PPDM, a compromise between specificity and secrecy is usually vital. Privacy requires hiding the underlying records of data prior to the analysis and precision requires the extraction of meaningful patterns amidst the disturbances of the data. PPDM has two primary methods of securing confidential information: cryptographic representations and such based on optimization algorithms. In modern industries, the emphasis is put on data protection when transporting and storing them in data warehouses. The most critical problem is the manipulation and retrieving of information in trained datasets with privacy. PPDM is a data mining adaptation of traditional algorithms that are productive in concealing sensitive data. The objectives that PPDM computes are:

- Stop gathering useful information.
- Having unhindered access and use of the non-sensitive information.
- Applying to vast data sets.
- Must be less exponentially difficult computationally.

PPDM methods often rely on data mining, encryption, and information concealment.

The knowledge systems such as decision tree algorithms are applied in clean data. This method has the benefit of being effective at managing big dataset volumes. Private information data collecting and processing a set of data divided among numerous private firms are the two main issues with PPDM. These algorithms’ goal is to obtain information from data warehouses while maintaining data confidentiality. Numerous PPDM techniques have been developed recently, however, none of them have indeed been standardized.

The remainder of this paper is organized as follows. Section "Literature survey" presents the literature survey, discussing existing privacy-preserving data mining techniques and their limitations. Section "Proposed methodology" describes the proposed methodology, including the K-means + + clustering, Flip-and-Rotation Perturbation (FRP), and AHP-based classification framework. Section "Results and discussion" provides experimental results and performance evaluation. Finally, Section "Conclusion" concludes the paper and outlines directions for future research.

**Contributions of the Study.**

The key contributions of this work are threefold:

1. A hybrid privacy-preserving framework that integrates clustering-based preprocessing with Flip-and-Rotation Perturbation to reduce information loss;
2. A novel application of Analytic Hierarchy Process as a structured decision model for classification on perturbed data; and
3. A comprehensive evaluation demonstrating improved privacy–utility trade-off compared to conventional fuzzy and probabilistic classifiers.

Unlike existing approaches that apply perturbation or classification independently, the proposed framework uniquely integrates clustering, transformation, and decision modeling into a unified pipeline. The use of K-means + + enables structure-aware grouping prior to perturbation, reducing distortion. The FRP technique preserves geometric relationships while enhancing privacy, and the AHP-based classification introduces an interpretable decision-making mechanism. This coordinated integration allows the model to achieve an improved balance between privacy preservation and classification performance compared to standalone methods.

## Literature survey

The matrix is transformed by a randomized transformation matrix before publication in order to protect data privacy. Randomized rotation is one of the popular perturbation methodologies for private information data categorization. As a result, numerous types of classifiers that are based just on feature extraction of the data can reach similar accuracy on the converted data as they do on the original information. This technique has the clear benefit of being able to keep the geometrical qualities of the data matrix. In^11^, the author describes a random rotation disturbance strategy for data categorization that protects anonymity. In further detail, we highlight the significance of classification-specific data about the information loss factor and offer a random rotational disturbance platform for data classification with privacy protection. Our method has two distinctive features. First, we note that several classification techniques make use of the datasets’ geometrical features, which may be maintained via geometrical rotations. We demonstrate that the three different classifier classes will consistently perform well on the rotation-perturbed dataset as they do on the original data. To overcome the issues with measuring privacy quality for multivariate perturbation, we secondly present a multi-column privacy model. The authors created a local optimum method using this measure to identify the best rotation disturbance in terms of confidentiality assurance. With the confidentiality model, we additionally examine naïve estimates and ICA-based reconstructive attacks. Our preliminary research demonstrates that the randomized rotation strategy may provide high levels of privacy while preserving precision. The authors of^12^ expand this concept to the case in which it is assumed that the data matrix is horizontally partitioned into a variety of sub-matrices and is owned by several parties. Each data bearer can individually and arbitrarily select a transformation matrix to change their specific data. The altered data is then sent by each party to a third party, who compiles it into a single dataset for data mining or other analytical needs. Researchers demonstrate that this type of plan may retain the accuracy of several classifications and clustering techniques used to the modified data as well as to the original data by preserving the geometric aspects of the data set. Instead of using mathematical techniques, this technique enables us to create effective centralized data mining methods that protect privacy. Such generalization is successful for data sets that have been vertically partitioned, according to experiments on actual data sets. The most important viewpoint among researchers is thought to be Privacy Preserving Data Mining (PPDM). Sharing and trading of privacy-vulnerable data for analysis has become more and more common as privacy-preserving data mining advances. Since protecting privacy is one of the most important parts of data mining. Various methods are used to protect privacy while keeping the true nature of the data under examination. The high-dimensional information in the proposed study is divided into several portions using the k-mean clustering approach, and each division is treated as a cluster. The difference between each new cluster and the mean of both cluster esteem is evaluated after the mean estimation of each cluster has been analyzed. In the following stage, the Ant Colony Optimization (ACO) technique is used to improve the clustered data. The Random Rotation Perturbation (RRP) technique is then used to disturb the optimized cluster particles, which makes it challenging to identify the values. The important parameters for randomizing and clustering are then maintained in the private cloud, while these disturbed results are kept in the public cloud. If we essentially put all sensitive data on private clouds, our technique would help to reduce the amount of storage in a cloud environment. The experimental findings show that the RRP method protects privacy better than any other available method^13^. A commonly used and approved Data Mining (PPDM) method used to establish a single degree of confidence in data miners is the data perturbation method. The issue of creating accurate models regarding aggregated without access to exact information or original records in individual data records is addressed by private information data mining. The Perturbation-Based PPDM technique randomly perturbs each value to protect data privacy before publication. This approach’s earlier works are not appropriate for single-level confidence in data miners. Our premise is taken into account in this study as we broaden the use of perturbation-based PPDM to multilevel trust (MLT-PPDM). The more trustworthy a data miner, the less perturbed duplicate may access privileges. A malevolent data miner would thus be able to access several copies of the same material in various ways, combining these copies to collaboratively deduce extra information about the original information that the data holder does not wish to share. Offering MLT-PPDM services presents the difficulty of preventing diversity assaults. Here, we attempt to overcome this difficulty by appropriately perturbing assignments across copies at various confidence levels. We demonstrate the effectiveness of our approach in preventing diversity assaults in light of our privacy policy. In other words, our method prevents data miners from accessing arbitrary collections of perturbed copies to jointly rebuild the original data more precisely than the best attempt utilizing any one copy in the community. Our method helps a data owner create disturbed copies of their data on demand by trust levels. This method gives information owners the most freedom possible^14^. Concerns about individual privacy arise now that corporations are utilizing clusters as a technique for data analysis. Data mining systems must protect sensitive and personal data found in criminal, medical, and financial records while also preventing privacy leaks. To safeguard the values of the underlying sensitive characteristic whenever sharing the information for clustering over centralized data, a private information clustering approach is presented in this research. The suggested approach is based on fuzzy logic and randomized rotation disturbance ideas. With this method, secret numerical characteristics are kept private without compromising the results’ accuracy. The results show that the suggested method is efficient and offers a workable strategy for striking a balance between privacy with reliability^15^. The most difficult problem we are now experiencing is the protection and confidentiality of the data, which increases the likelihood of being easily destroyed as internet and web application usage grows more quickly. Condensation, randomization, tree structures, and other privacy-preserving approaches have limits in that they are unable to maintain a suitable balance between the value of the data and privacy and thus may result in privacy violations. An additive rotation perturbation method for privacy-preserving data mining is presented in this research (PPDM). In the proposed study, multiple datasets from the UCI Machine Learning Repository were gathered and secured under privacy preserving data mining using a New Additive Rotational Perturbation Technique. The strength of the suggested approach, which is evaluated by using the DoV (Difference of Variance) technique, is demonstrated experimentally to be strong for all databases^16^. Data sources are really difficult work for the algorithms. It processes. One of the most crucial data mining techniques is classification, and in this research, we employed an ensemble model to categorize the data to increase classification accuracy. After the subsequent process of reconstructing the primary data from the disturbed data, randomization is introduced to the sensitive privacy data. The variety in the information is preserved using Principal Component Analysis (PCA). Rotating transformation can enhance the number of base classifiers and boost the ensemble classifier’s accuracy. In this article, researchers analyze the use of a Z score-normalization approach, a load line plot, and a rotation perturbations strategy for PCA to locate eigenvectors in streaming mining^17^. Big data security and privacy have recently grown to be significant challenges, necessitating the use of privacy-preserving data mining approaches to preserve the trade-off between data value and privacy. Web mining is the technique of data-mining methods for mining the web data. The majority of users desire to retain tight anonymity when using web apps and engaging in online activities, which gives rise to security difficulties on the web. Condensation, randomness, tree structures, and other traditional methods are employed to protect privacy. Keeping a delicate balance between data value and privacy as well as the scale issue are the constraints of the current systems. By using privacy-preserving methods like perturbations, encrypting, machine learning, etc., the transmitted data may be protected and categorized. Here, the altered data streams are classified with the help of the convolution neural network algorithm Naive Bayes, which offers a high level of accuracy. The UCI web mining databases are gathered for this proposed study. Fuzzy C-Means (FCM) are used for clustering, the Flip and Rotation Perturbation (FRP) approach is used for data renovation, and the Nave Bayes classifier is used to classify perturbed data. By adopting a holdout method for data isolation during the training and testing stages, the classifier is enhanced. Measured and contrasted to those of the current approach are the classifier’s level of accuracy, computational burden, and margin of error. The comparison demonstrates the recommended method’s success. This suggested system was created using MATLAB 2018a ^18^. Certain issues arise when we collect private, confidential, and corporate information using data mining programs. The abuse of personal data results in privacy attacks. In this research, data transformation techniques for fuzzy-based data in the area of private information clustering are given for use in central data environments. In the first instance, a strategy for transforming fuzzy data is suggested, and several tests are run by altering the fuzzy membership functions, such as the Z-shaped fuzzy membership function, the trapezoidal fuzzified function, and the Gaussian fuzzification function. In the second case, a hybrid strategy that combines the first case’s stated fuzzy data processing methodology with random rotation perturbation is provided (RRP). The findings of the experiment supported the claim that the hybrid strategy enables the best outcomes for each member function ^19^.

Recent advancements in privacy-preserving data mining emphasize the importance of balancing data utility and confidentiality through adaptive anonymization strategies. Differential privacy–based approaches, such as correlation-aware mechanisms for medical datasets, highlight the importance of preserving attribute relationships while ensuring privacy guarantees^20^. Additionally, comprehensive reviews on anonymization techniques in big data environments underline the effectiveness of perturbation and clustering-based hybrid models in maintaining analytical utility^21^. Emerging research on stream data anonymization using in-memory processing further demonstrates the need for scalable and efficient privacy-presing frameworks^22^. Motivated by these developments, the proposed FRP-AHP model integrates clustering and perturbation to achieve an improved privacy–utility trade-off in structured datasets.

The datasets used in this study are publicly available benchmark datasets obtained from the UCI Machine Learning Repository. All experiments were repeated multiple times, and average performance metrics were reported to ensure reproducibility. Experiments were conducted on two publicly available benchmark datasets from the UCI Machine Learning Repository: the Cleveland Heart Disease dataset and the Adult Income dataset. Both datasets contain mixed numerical and categorical attributes suitable for evaluating privacy-preserving data mining techniques.

## Proposed methodology

**Fig. 2:**
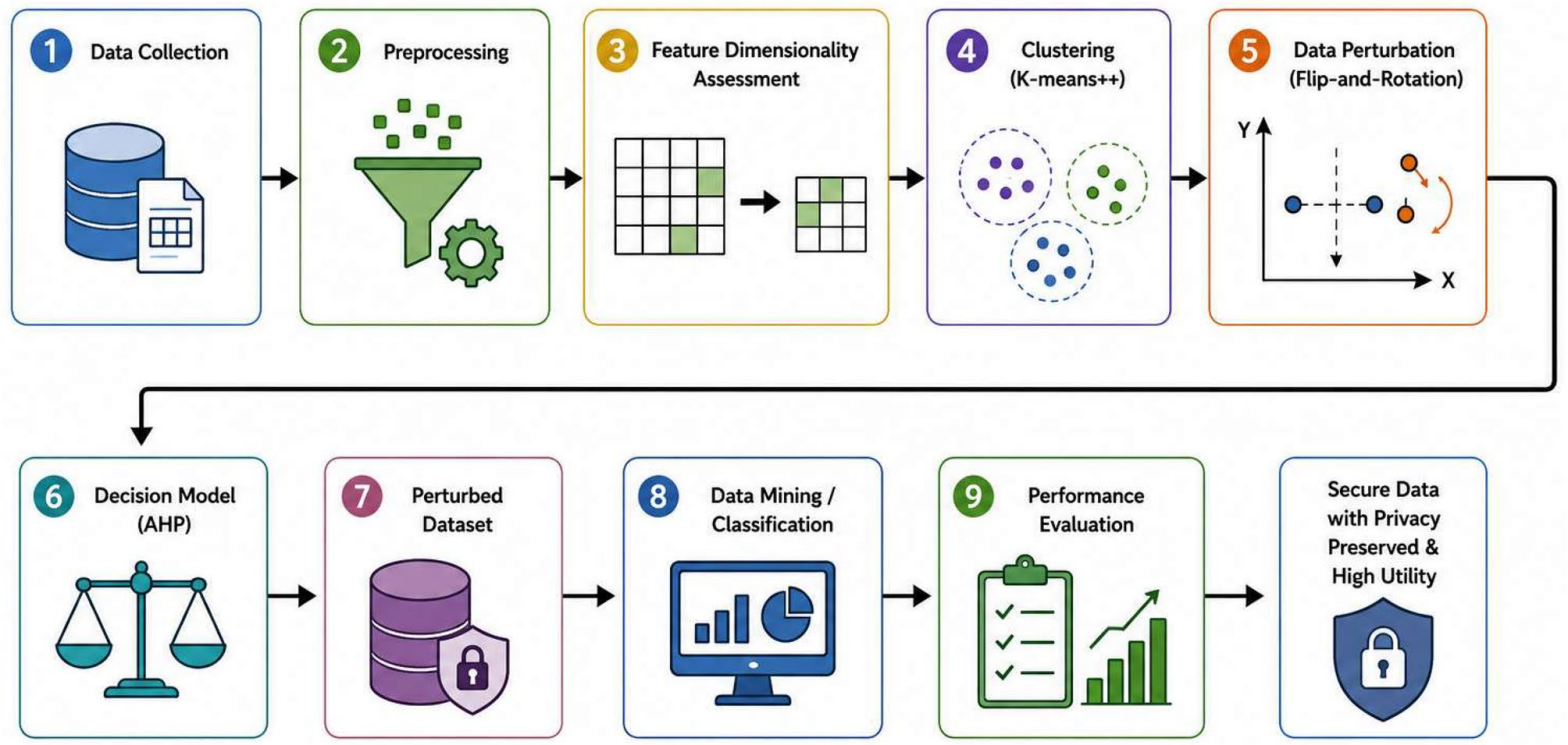
Proposed Data protection scheme steps.

Data mining methods use patterns for finding and extracting information. These approaches also include inferring methods and patterns of identification that are frequently used in data analysis. The raw database is initially normalized before the K-means +  +  technique is used to accomplish clustering^23^. The data would then be perturbed using the flip and rotation approach. Then is used to classify this altered data. Here, the holdout approach is used to frame the training and testing data. Measures of classification accuracy and time use are produced, and they are contrasted with those of other available techniques. Figure 2 depicts the suggested methodology’s overall process flow. The alteration of the data enhances the security of the data while preserving privacy.

The suggested methodology’s overall workflow and the steps to accomplish data modification and categorization are:

1. Raw data selection
2. Clustering using K-means +  +  algorithm
3. Flip and Rotation Perturbation (FRP) and implement PPDM
4. Holdout approach for the separation of data into training and testing phases at 0.10% v
5. AHP Classification
6. Measurement of performance analysis

To prevent the release of raw data, it is standard practice to require privacy preservation from various user roles throughout the data preparation step. In principle, two sorts of user roles guard against the release of confidential information during the preparation stage of data. Before using privacy-preserving approaches, data pre-processing entails several procedures to address noise, outliers, and missing information. The following is a description of the process:

A smoothing function S(X), such as median filtering, can be used to minimize noise in data X.

1 $${X}_{smooth}= S(X)$$

Outliers in data X are identified using the Z-score method:

2 $${Z}_{i}=\frac{{X}_{i}-\mu }{\sigma }$$

where *μ* is the mean and *μ* is the standard deviation of X.

Mean imputation is one method for imputing missing values in data X:

3 $${X}_{imputed}= mean({X}_{observed})$$

These steps contribute to enhancing the overall performance and reliability of the privacy-preserving data mining approach. A part of the supervised machine learning technique, k-means +  + clustering manages the data according to categories. In principle, the data perturbation technique may be used to execute data splitting, data modification, data limitation, and information governance rights protection^24^. In malware attacks, the information is altered in a manner that safeguards the confidentiality of the given dataset. This information disturbance executes data rotation and shuffling operations to enhance the outcomes of the privacy-preserving technique^25^. Provided by, the flip procedure in data perturbation ().

The Flip-and-Rotation Perturbation (FRP) process is defined as:

4 $${\text{X' = (X }} \odot {\text{ F) R }}$$

where F is a binary flipping matrix that selectively inverts feature values, ⊙ denotes element-wise multiplication, and R is an orthogonal rotation matrix that preserves the geometric structure of the data.

The perturbed dataset X’ is then classified using the Analytic Hierarchy Process (AHP), formulated as:

5 $$y = argmax\__{i} (W\_i^{ \wedge } TX^{\prime})$$

where W_i represents the priority weight vector obtained from pairwise feature comparisons corresponding to class i.

**Fig. 3:**
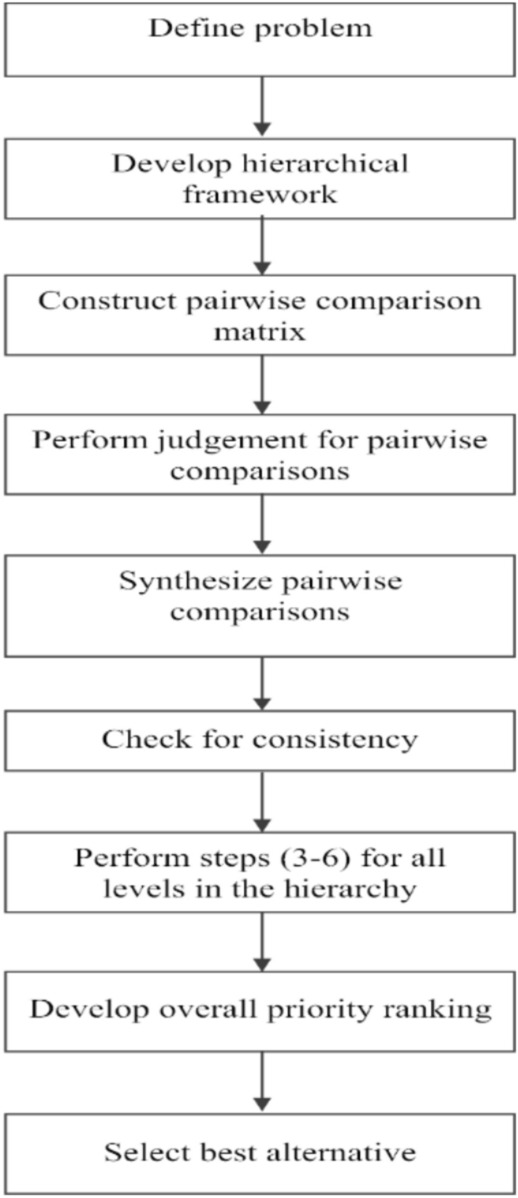
Flowchart for AHP.

The trade-off between data usefulness as well as data protection is maintained by the FRP approach, which creates the disturbed data. Electroencephalography (EEG) signal values are included in the ESDS for recognition, and by finding the class value of the classification, it distinguishes between the seizure and no seizure categories. Here, the data and information are perturbed using the K-means +  + clustering and FRP approaches. This method protects user data without altering the original content, and the conserved clustering enhances data utilization without jeopardizing user privacy. The disturbed data is then created by shuffling the input data. The matrix used to organize the data in the database is made up of clustered data perturbation. After that, the AHP approach is used to classify the altered data is visually presented in Fig. 3.

The Analytic Hierarchy Process (AHP) is employed as a structured decision-making mechanism to classify perturbed data samples. Feature importance is determined through pairwise comparisons using a normalized comparison matrix. Priority vectors are computed using eigenvalue decomposition, yielding feature weights that reflect relative importance. Each perturbed instance is evaluated using weighted aggregation, and the class label corresponding to the highest score is assigned to the sample.

Analyzing the characteristics of the research is crucial to prevent it from being accessed by the wrong individuals. In this viewpoint, we primarily concentrate on three goals. In social networking sites, three types of privacy have been protected: User characteristics about age, gender, birthday, region, and hobbies should be saved^26,27^. User data about links between friends or other users should also be well-maintained. Customer characteristics about node data should also be maintained. Data owners, or network operators, are people or organizations who are required to give data collectors (who keep data storage computers with potentially sensitive information) their initial source data e.g., academic records of students, and financial transcripts of customers. The fundamental problem for network operators is their ability or lack thereof to manage the level of information sensitivity that they give data collectors. The idea is that information gatherers are not trustworthy^28^. To safeguard confidentiality disclosure and obtain proper compensation for the potential loss of private information, the data provider safeguards that improve security during data analysis results and data transmitting to data warehouse servers significantly aim to conceal their confidential material or prevent unauthorized access. Data suppliers consider their data to be very confidential, hence they are unable to divulge any information^29^. They try to take appropriate precautions to protect sensitive data and deny the order to divulge such data. Since data owners are aware of the worth of their information to data providers, they choose never to divulge personally identifiable details (private). As a result, data providers falsify the information they send to data providers to make it harder to discover the truth. Data providers could be prepared to provide part of their confidential communications in exchange for certain perks, including better services or financial gain. They need to know how to bargain with the data collector to get adequate compensation for any possible privacy losses^30^.

If data providers are unable to restrict access to their private data or engage in a commercial transaction that benefits both parties, data collectors may distort the data that the data owners have provided in ways that make it difficult to ascertain the truth in Fig. 3.

The process of clustering, a sort of unsupervised classification, comprises finding groupings of valued items (assumptions) that are comparable to each other (clusters). A certain amount of fine information is necessarily lost when data is represented by a small handful of clusters. Nevertheless, simplifying is possible. In other words, items from distinct clusters have less in common when a complete data set is divided into groups. So groupings to which a people belong do not always have to be comparable or predetermined. The statistics may also show that the groups have unrecognized relationships^31^. Consequently, clustering is also known as automated object categorization. Individuals’ perceptions of the unprocessed training data used to symbolize these categories have an impact on how many clusters are formed. So because the term “cluster” is not clearly defined, the number of clusters is the primary limitation in clustering^32^. To locate coherent groupings in huge data sets, several methods have already been presented, and each technique adheres to a certain set of principles. Users will choose the most appropriate technique most effectively if they grasp the issue and the corresponding data kinds. By analyzing the connection between the characteristics and the classifications of the objects in the training dataset, classifying is frequently used as a supervised learning approach to build a model that can predict the category labels (the class label attributes). As a result, existing algorithms employ various techniques to look for these links. A data mining technique called classifying assigns each item to a predetermined group of classes or groups its proper class based on its properties. The classification technique is the basic sort of classifying and includes predicting one of two target classes. Multiclass targets, on the other hand, have more than 2 different classes. In a manner similar, classifying processes involving two classes leads to complex methods. There are two phases in the data classification process. The training portion comes first. In this stage, a model or classifier is built to define a preset collection of data classes, which will later be employed to classify data. Rule-based approaches, induction of decision tree methods, neural networks, memory-based learners, support vector machines, and Bayesian networks are the most often used techniques for classifying data mining problems^33^. Numerous algorithms have been devised by taking into account the aforementioned qualities. There are three main areas where confidentiality has to be protected. It’s them,

- Privacy node
- Private attributes
- Privacy Link

### Privacy model

In this work, privacy preservation is focused on structured tabular datasets commonly encountered in healthcare and enterprise analytics. Attributes are categorized as sensitive or non-sensitive based on their potential to reveal personal or confidential information. Perturbation is a method of providing privacy for sensitive attributes without loss of any statistical properties that would be needed by data mining algorithms.

The Flip-and-Rotation Perturbation (FRP) technique ensures that sensitive attribute values are converted in a way so as to make them not directly re-constructible and yet their inter-attribute relationships are known to do inter-attribute classification. This method prevents unnecessary information loss, and aids in correct downstream learning exercises.

### Computational complexity analysis

The computational complexity of K-means +  + clustering is O(nkd), where nnn is the number of instances, k the number of clusters, and ddd the feature dimension. The Flip-and-Rotation Perturbation incurs)O(nd) complexity, while AHP-based classification operates in O(d^2 + nd)). Overall, the proposed framework scales efficiently for medium to large datasets.

Random rotation–based perturbation has been widely adopted for privacy-preserving data classification due to its ability to retain geometric properties of datasets while obscuring sensitive values. Prior studies demonstrate that classifiers relying on distance or distributional characteristics can achieve comparable performance on rotated data as on original datasets. Furthermore, extensions of this approach for distributed and partitioned datasets allow independent transformation by multiple data owners, ensuring privacy without centralized exposure of raw data. However, these methods often lack mechanisms to optimize the trade-off between privacy and classification accuracy, motivating hybrid approaches such as the one proposed in this work.

**Algorithm 1:**
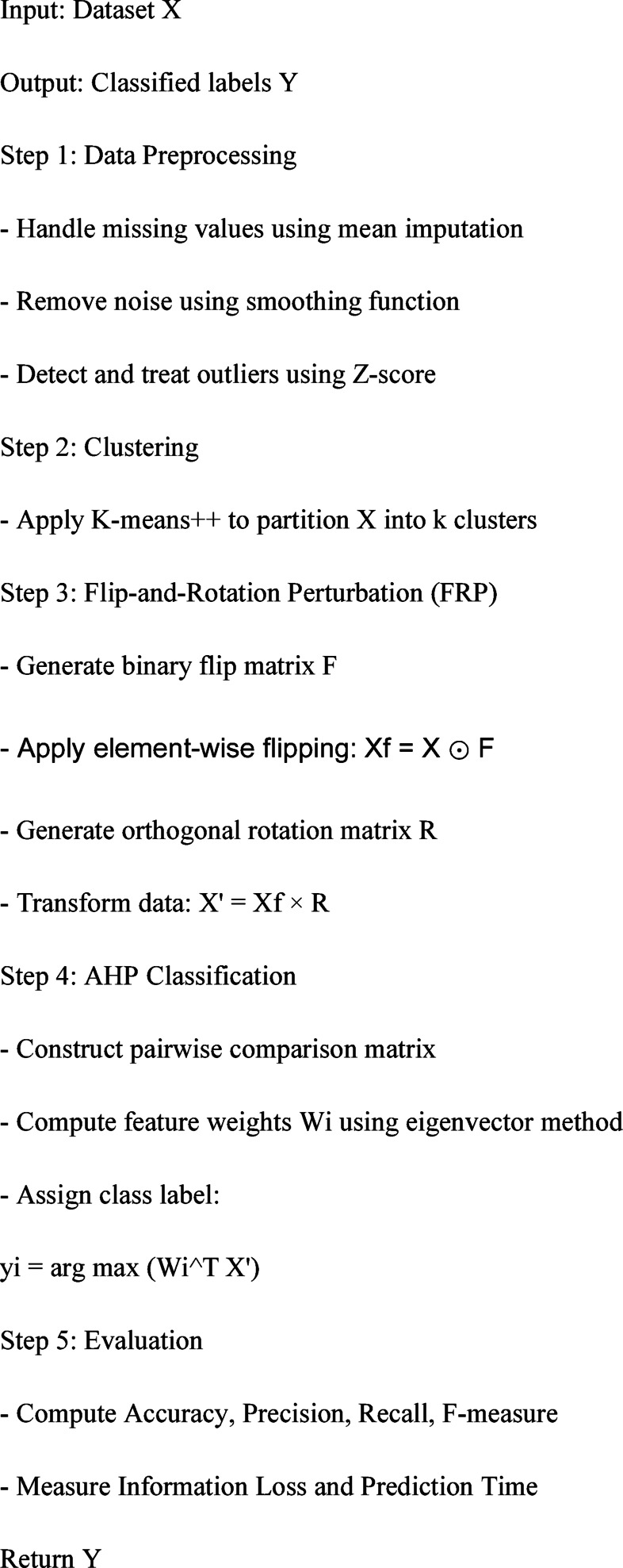
FRP-AHP Privacy-Preserving Data Mining Framework

## Results and discussion

In our experiments, we generated a dataset comprising 100,000 records. From this dataset, 66,000 records were randomly selected for training, and the remaining 34,000 records were designated for testing purposes. We employed the WEKA data mining software to implement and evaluate our proposed classifiers using this reconstructed dataset^34–43^. The experiment results, including performance metrics, were reported based on the evaluation conducted on the test data subset. In this part, we compare the outcomes of our suggested scheme to earlier methodologies. AHP approaches and two dependent on the expected were applied. The experimental configuration and dataset characteristics used to evaluate the proposed FRP–AHP framework are summarized in Table 1.

**Table 1:** Experimental Dataset and Evaluation Setup.

Parameter	Description
Dataset Source	UCI Machine Learning Repository
Datasets Used	Cleveland Heart Disease Dataset, Adult Income Dataset
Total Records	100,000
Training Records	66,000
Testing Records	34,000
Data Preprocessing	Noise removal, mean imputation, outlier handling
Clustering Technique	K-means + +
Privacy Technique	Flip-and-Rotation Perturbation (FRP)
Classification Method	Analytic Hierarchy Process (AHP)
Evaluation Tool	WEKA
Performance Metrics	Accuracy, Precision, Recall, F-measure, Prediction Time, Throughput, Information Loss

Accuracy is measured as the proportion of correctly categorized cases in the dataset. In mathematical terms, it is calculated as:

$$Accuracy =\frac{No.\, of. \, Correct\, Instances}{Total\, count\, of\, predictions}\times 100$$

Precision is the percentage of true positive predictions among all positive predictions made by the model. The calculation is as follows:

$$Precision =\frac{True\, positives}{True\, Positives + False\, Positives}\times 100$$

Recall calculates the fraction of genuine positive predictions among all real positive instances in the dataset. It’s computed as:

$$Recall =\frac{True\, positives}{True\, Positives + False\, Negatives}\times 100$$

The harmonic mean of precision and recall is F-measure that gives a justifiable measure of performance of the model. It is calculated by the following formula:

$$F-measure=\frac{2\times Precision\times Recall}{Precision+Recall}\times 100$$

Comprehensively, the metrics indicate that the proposed system is useful in reliably classifying the instances with a balance between reducing false positive and false negative errors, which leads to high-quality classification outcome. The experiment revealed that the AHP point quickly NB and algorithms in regards to accuracy, precision, recall, and f-score, as shown in Table 2.

**Table 2:** Performance Evaluation of Classification Algorithms.

Method	True positive rate (%)	Accuracy (%)	Precision (%)	Recall (%)	F-measure (%)
General Fuzzy	72.1	76	83.45	56.98	67.54
NB	80.25	83.33	76.66	56.5	64.58
Proposed	99.1	99.45	99.4	99.2	99.12

The privacy in the proposed system is defined by the comparison of the shared sensitive pattern of both original dataset D and the perturbed dataset D. This analogy is essential in explaining the extent of ensuring sensitive information is taken care of or disclosed through manipulating of data. Also, the level of privacy can be assessed by means of the measuring of the level of the hiding failure (HF) that is the percentage of the restricted patterns which are found in the sanitized dataset D’ as compared to the number of the restricted patterns which are found in the original data D. The play-book of how to hide failure (HF) can be expressed as follows,

$$HF =\frac{Rp(D{\prime})}{Rp (D)}$$

where, $Rp(D{\prime})$ reflects the number of restrictive patterns detected in the sanitized dataset D, while *Rp(D)* represents the number of restrictive patterns found in the original dataset D. This measurement helps evaluate the effectiveness of the privacy-preserving mechanisms used in the proposed system, assuring adequate security of sensitive information while maintaining data utility. Findings of the above mentioned comparison are that our presented work is the best among other available methods in the form of generalized fuzzy and NB.

The many data mining algorithms that we compared in our study are represented by these categories. Naive Bayes stands for the Naive Bayes classifier, and General Fuzzy probably alludes to a generalized fuzzy inference system. Our innovative privacy-preserving data mining approach, which combines machine learning and perturbation techniques, is represented by the category labeled Proposed.

**Fig. 4:**
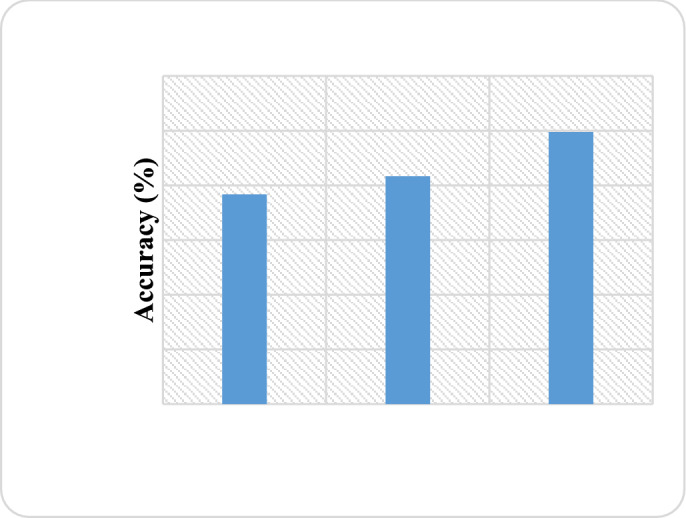
Comparison on Accuracy.

The accuracy results of various tweets labeled as “private” and "non-private" are shown in Fig. 4. The overall accuracy rate for categorization is high (99.4%). Since the system is more complicated and exhibits considerable fluctuations, the accuracy is low for both the general fuzzy inference system and Nave Bayes techniques. The accuracy measure is utilized to further compare the proposed system’s classification performance to the earlier methods.

**Fig. 5:**
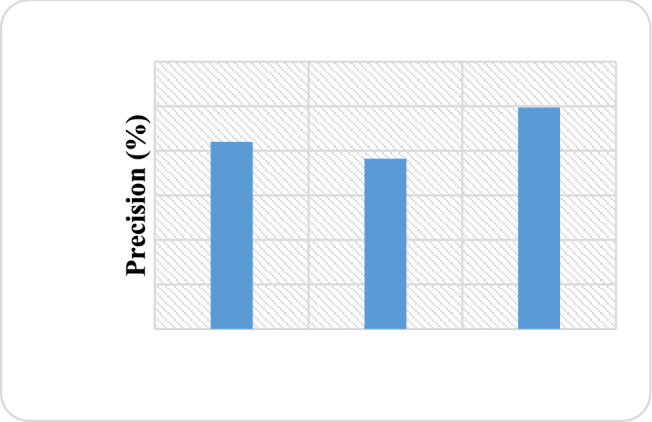
Comparison of Precision.

The average precision rates for several methods, including generic fuzzy and Naive Bayes techniques, are compared in Fig. 5. Although the accuracy is increased, this is not the case for general fuzzy and naive Bayes algorithms, which use a single node design. When the quantity of tweets rises, so does the training time. Because of this, the suggested AHP achieves a greater level of precision than naive Bayes and broader fuzzy logic systems. The accuracy and recall rates are typically utilized as conventional criteria to analyze as well as assess the categorization accuracy.

**Fig. 6:**
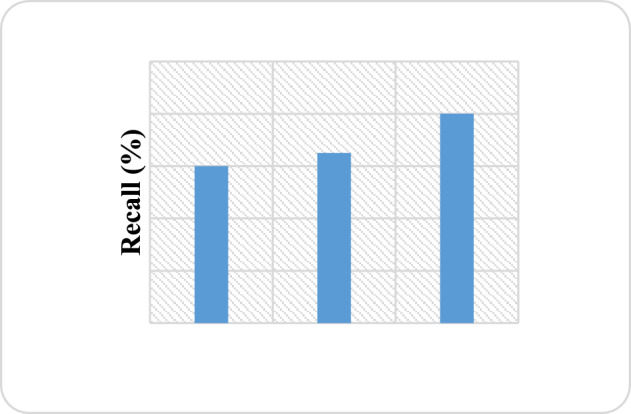
Comparison of Recall.

The graph contrasts the typical recall rates for users across all categories of tweets. The recall rates of our suggested method are greater than those of the conventional fuzzy and Naive Bayes techniques. To calculate the F1-score, combine the accuracy rate and recall rate in Fig. 6. As a general performance measure of categorization quality, the F1 value is employed. A stronger classifying effect and greater classification accuracy are indicated by a higher F1 value.

**Fig. 7:**
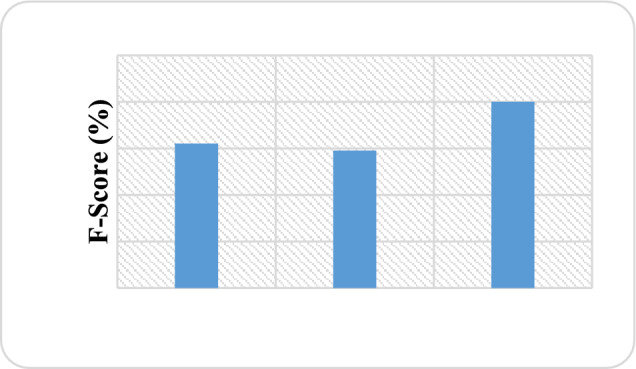
Comparison of F-Score.

The F1 results for tweet samples are compared in Fig. 7 with those from several earlier categorization methods. Additionally, the technique recommended in this research improves the performance of the classifier by using the AHP system to optimize the performance of the system.

A thorough comparison of the proposed approach to the existing methods (General Fuzzy and Naive Bayes) is shown in Comparison Table 3 across several important parameters.

**Table 3:** Comparison of existing and proposed system.

Method	Prediction Time (s)	Throughput (%)	Information Loss (%)
General Fuzzy	0.6	80	10
NB	0.9	70	15
Proposed	0.4	90	5

**Fig. 8:**
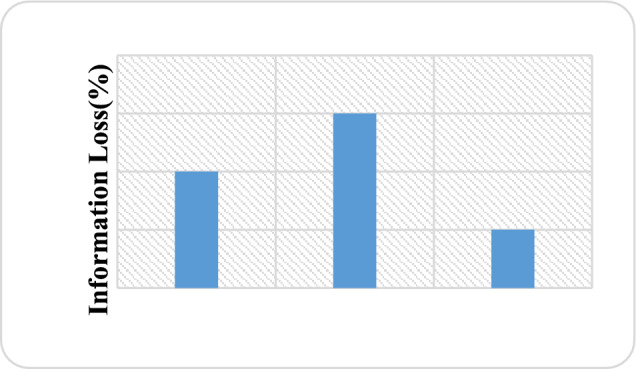
Comparison of existing and proposed system.

In observance of Fig. 8, the proposed method produces a substantially lower information loss of 5% as opposed to 10% for General Fuzzy and 15% for Naive Bayes. This suggests that the suggested method more effectively protects the original data’s usefulness while maintaining privacy.

**Fig. 9:**
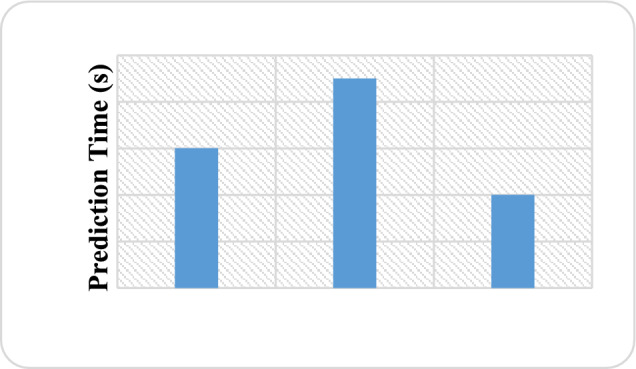
Comparison of existing and proposed system.

In terms of Efficiency Metrics in Fig. 9, the methodology used is more efficient compared to General Fuzzy and Naive Bayes, the proposed method yielded a prediction time (0.4 s) more than General Fuzzy (0.6 s) and Naive Bayes (0.9 s). This faster forecast time makes the system more efficient when it is handling input on a real time basis.

**Fig. 10:**
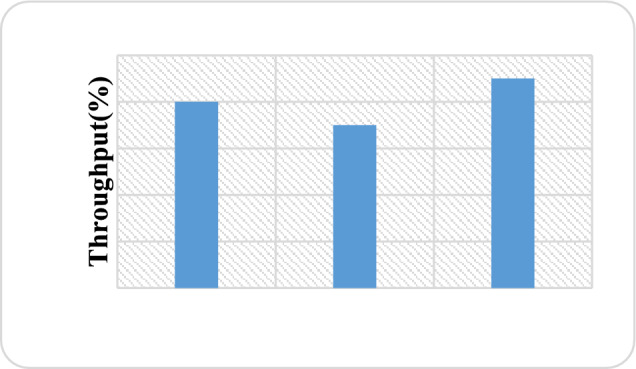
Comparison of existing and proposed system.

Figure 10 indicates that the proposed approach performs better than General Fuzzy and Naive Bayes by 80 percent and 70 percent respectively in respect to Scalability Metrics. Such increased throughput helps to show the extent to which the proposed solution can handle greater amounts of data and hence is more scalable to larger variety of applications. Overall, the comparative analysis reveals the advantages of the proposed strategy to increase the privacy protection, effectiveness in prediction operations, and scalability to such a large set of data. The obtained results prove the effectiveness and applicability of the suggested approach to real-life data mining and analysis problems.

The structured benchmark data was evaluated experimentally. Accuracy, precision, recall, F-measure, prediction time, throughput and information loss measures were used to evaluate the performance. The developed strategy was always better than Naive Bayes and generalized fuzzy models in all assessment criteria.

## Conclusion

This paper introduced a perturbation-based privacy preserving data mining system that combines clustering, Flip-and-Rotation perturbation as well as AHP-based classification. The suggested technique is a good compromise between data utility and privacy as it can decrease dimension before perturbation and use systematic decision analysis to make classification. The performance of the experiment has proven to be better than the traditional fuzzy and naive bayes classifiers with reference to accuracy, efficiency, and information loss. The results of the research show that the approach proposed is indeed feasible in terms of secure and accurate data mining in sensitive application areas. PPDM will be performed using blockchain-based techniques of deep learning in the future.

## Data Availability

The datasets used and/or analysed during the current study available from the corresponding author on reasonable request.
